# Two New Acridone Alkaloids from *Glycosmis macrantha*

**DOI:** 10.3390/molecules16064401

**Published:** 2011-05-27

**Authors:** Maizatul Akmal Yahayu, Mawardi Rahmani, Najihah Mohd Hashim, Muhammad Aizat Mohd Amin, Gwendoline Cheng Lian Ee, Mohd Aspollah Sukari, Abdah Md Akim

**Affiliations:** 1 Department of Chemistry, Universiti Putra Malaysia, 43400 UPM, Serdang, Selangor, Malaysia; 2 Department of Biomedical Science, Universiti Putra Malaysia, 43400 UPM, Serdang, Selangor, Malaysia

**Keywords:** *Glycosmis macrantha*, macranthanine, 7-hydroxynoracronycine, atalaphyllidine

## Abstract

Extraction and chromatographic separation of the extracts of dried stem barks of *Glycosmis macrantha* lead to isolation of two new acridone alkaloids, macranthanine (**1**) and 7-hydroxynoracronycine (**2**), and a known acridone, atalaphyllidine (**3**). The structures of these alkaloids were determined by detailed spectral analysis and also by comparison with reported data.

## 1. Introduction

Plant of the genus *Glycosmis* of the family Rutaceae, are widely distributed throughout the India-Malayan region. They are small trees, commonly found on limestone hills and near coastal areas. Fourteen of the species can be found in Peninsular Malaysia and several members of the genus were traditionally used for the treatment of various diseases such dysentery, fever cough, jaundice, rheumatism, eczema and skin diseases [1,2]. Previous reports indicated that the genus is a rich source of various classes of compounds such as flavonoids, alkaloids and sulphur-containing amides [3,4,5,6,7,8]. Some of these compounds were reported to exhibit biological activity, particularly the sulphur-containing amides, which showed strong anticancer properties towards a number of cell lines and antitrypanosomal activity [9,10]. In this article we wish to report the isolation and structural identification of three acridone alkaloids, including two new compounds, from the dried stem barks of *Glycosmis macrantha.*

## 2. Results and Discussion

**Figure 1:**
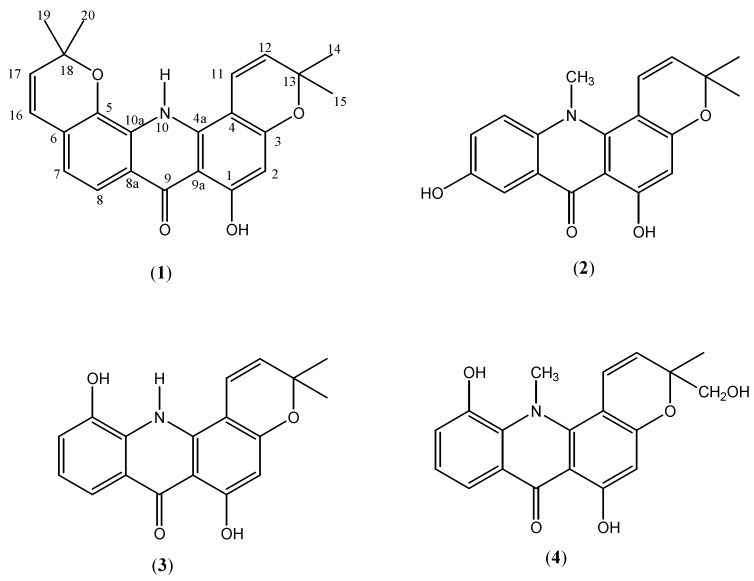
Structures of acridone alkaloids.

Silica gel chromatographic separation of the hexane extract with mixture of solvent systems yielded macranthanine (**1**, 24 mg) as yellowish needle-shaped crystals. Similar repeated chromatographic separation of the methanol extract gave 7-hydroxynoracronycine (**2**, 118 mg) also as yellowish needles, together with atalaphyllidine (**3**, 108 mg) as a yellow powder (Figure 1).

Macranthanine (**1**) formed yellowish needle-shaped crystals with m.p. 216-218 °C after recrystallisation from hot chloroform. The UV spectrum exhibited abrorptions at λ_max_ 303, 239 nm, typical characteristic of acridone skeleton [12], coupled with the appearance of a prominent band at 1642 cm^−1^ in the IR spectrum that suggested the presence of a chelated carbonyl group. This was further substantiated by the appearance of a very downfield signal at δ 15.10 for the chelated hydroxyl group in the ^1^H-NMR. A molecular ion peak at *m/z* 375 was noted in the EIMS spectrum, corresponding to a molecular formula of C_23_H_21_NO_4_. The ^1^H-NMR resonances revealed the existence of two 2,2-dimethylchromene rings. A set of doublets at δ 5.65 and 6.72, each with a 10.08 Hz coupling constant, were assigned to protons at H-12 and H-11 (Table 1). Another set of doublets with identical coupling constants of 10.12 Hz at δ 5.82 and 8.24 were due to protons at H-17 and H-16. The four methyl groups of the two chromene rings appeared as a twelve proton singlet at δ 1.43. In the aromatic region, the *ortho*-coupled doublet signals at δ 7.18 and 7.33 (each 1H, *d*, *J* = 8.28 Hz) and a lone singlet at δ 6.20 assigned for H-2 were observed. Another low field singlet at δ 11.03 attributed to proton attached to the nitrogen atom was also noted. The attachment of these protons to their respective carbon atoms were noted in the HMQC spectrum and the HMBC spectrum provided the ^2^*J* and ^3^*J* relevent correlations.

**Table 1:** ^1^H-NMR and ^13^C-NMR spectral data of macranthanine (**1**), 7-hydroxynoracronycine (**2**) and 5-hydroxynoracronycine alcohol (**4**).

No	(1)(400/100 MHz, (2) acetone- *d*_6_)			(2) (400/100 MHz, DMSO- *d*_6_)			(4) (500/125 MHz, acetone- *d*_6_) [13]	
	δ_H_	δ_C_	HMBC	δ_H_	δ_C_	HMBC	δ_H_	δ_C_
1-OH	15.10 ( *s*, 1H)	160.2	C_4_, C_9a,_ C_1_	15.50 ( *s*, 1H, -OH)	159.5	C_9a_	14.42 ( *s*)	165.5
2	6.20 ( *s*, 1H)	91.7	C_3_, C_9a_, C_1_	6.49 ( *s*, 1H)	91.4	C_4_, C_9a_, C_1_	6.10 ( *s*)	98.2
3	-	159.6	-	-	158.8	-	-	162.2
4	-	102.5	-	-	101.3	-	-	103.3
4a	-	142.9	-	-	143.6	-	-	148.8
5	-	148.8	-	7.75 ( *d*, *J* = 9.25 Hz, 1H)	118.1	C_7_, C_10a_,	OH	149.4
6	-	119.9	-	7.35 ( *dd*, *J* = 9.25, 2.76, Hz, 1H)	124.7	C_5_, C_10a_	7.33 ( *d*, *J* = 7.6 Hz)	120.9
7	7.18 ( *d*, *J* = 8.28 Hz, 1H)	125.1	C_6_, C_10a_, C_5_	9.95 ( *s*, 1H, -OH)	152.6	-	7.21 ( *t*, *J* = 7.6 Hz)	124.2
8	7.33 ( *d*, *J* = 8.28 Hz, 1H)	118.4	C_8a, _C_5_	7.60 ( *d*, *J* = 2.76 Hz, 1H)	108.1	C_8a_, C_6_	7.78 ( *d*, *J* = 7.6 Hz)	117.1
8a	-	115.0	-	-	135.9	-	-	125.8
9	-	184.0	-	-	179.2	-	-	182.9
9a	-	105.8	-	-	104.1	-	-	107.6
N-H	11.03 ( *s*, 1H)	-	-	3.79 (s, 3H, N-Me)	34.5	C_10a_, C_4a_	3.84 ( *s*, N-Me)	49.1
10a	-	137.8	-	-	121.1	-	-	138.1
11	6.72 ( *d*, *J* = 10.08 Hz, 1H)	127.2	C_13_, C_3_	6.65 ( *d*, *J* = 10.12 Hz, 1H)	115.3	C_13_	6.82 ( *d*, *J* = 10 Hz)	123.0
12	5.65 ( *d*, *J* = 10.08 Hz, 1H)	116.7	C_13_, C_4_	5.72 ( *d*, *J* = 10.12 Hz, 1H)	127.6	C_13_, C_4_	5.66 ( *d*, *J* = 10 Hz)	121.9
13	-	78.1		-	77.9	-	-	80.6
14	1.43 ( *s*, 3H)	27.4	C_11_, C_13_	1.43 ( *s*, 3H)	28.3	C_15_, C_13_, C_12_	1.47 ( *s*)	22.5
15	1.43 ( *s*, 3H)	27.4	C_11_, C_13_	1.43 ( *s*, 3H)	28.3	C_14_, C_13_, C_12_	3.64/3.76	67.5
16	8.24 ( *d*, *J* = 10.12 Hz, 1H)	122.3	C_18_, C_5_					
17	5.82 ( *d*, *J* = 10.12 Hz, 1H)	131.8	C_18_, C_6_					
18	-	75.6	-					
19	1.43 ( *s*, 3H)	28.5	C_17_, C_18_					
20	1.43 ( *s*, 3H)	28.5	C_17_, C_18_					

The ^13^C-NMR and DEPT spectra confirmed the presence of twenty three carbons, consisting of four methyl, seven methine, six quarternary, five oxygenated tertiary and one carbonyl carbon atoms. The carbonyl group and the two carbons attached to N-H (C-10a, C-4a) appeared at δ 184.0, 137.8 and 142.9, respectively, while the chemical shifts of the five oxygenated carbons (C-1, C-3, C-5, C13 and C-18) were observed to occur at δ 160.2, 159.6, 148.8, 78.1 and 75.6. The aromatic singlet at δ 6.20 exhibited correlations with oxygenated carbon at C-3 (δ 159.6), C-1 (δ 160.2) and quarternary carbon at C-9a (δ 105.8). Similarly, the chemical shifts at δ 7.18 showed ^3^*J* and ^2^*J* correlations with C-5 (δ 148.8) and C-6 (δ 119.9) which supported the attachment of the prenyl group to the aromatic ring through the oxygen atom. From these spectral data, the structure of this new compound was established as shown and the compound was given the trivial name macranthanine (**1**).

Further chromatographic separation of the methanol extract yielded 7-hydroxynoracronycine (**2**) that was recrystallised from hot chloroform to give yellowish needle-shaped crystals with m.p. 261-263 °C. After comparison of the spectral data with various references we believe that 7-hydroxy-noracronycine (**2**) is also a new compound. The UV spectrum also revealed the existence of an acridone basic skeleton with absorptions observed at 308, 296 nm. The IR spectrum is similar to that of **1** with the presence of strong absorptions at 1636 and 3365 cm^-1^ due to chelated carbonyl and hydroxyl functionalities, respectively. The molecular formula of the compound was derived as C_19_H_17_NO_4_ based on EIMS with molecular ion peak at *m/z* 323. The ^1^H-NMR revealed the presence of seventeen protons, including the two singlets, one integrating for three protons and the other for six protons, occurring in the high field region assigned to the three methyl groups. The characteristic signals of a 2,2-dimethylchromene ring could be seen clearly by the occurrence of a pair of doublets at δ 6.65 (*d*, *J* = 10.12 Hz) and 5.72 (*d*, *J* = 10.12 Hz) and a six protons singlet δ 1.43 for the two methyl groups (Table 1). These data are similar to the spectral data of 5-hydroxynoracronycine alcohol (**4**) except for arrangement of protons in the aromatic ring C [13]. An ABX system was observed in the ring with the occurrence of two doublets at δ 7.60 (*d*, *J* = 2.76 Hz), 7.75 (*d*, *J* = 9.25 Hz) and a doublet of doublet at δ 7.35 (*dd*, *J* = 9.25 Hz, 2.76 Hz). The HMBC correlations spectrum further substantiated this assignment. In (**4**), the protons in the ring were arranged in an ABC system [13]. Further evidence to support the proposed structure could be seen in the ^13^C-NMR and DEPT spectra with the observation of three primary, six methine, four oxygenated, six quarternary and a carbonyl carbons. The chelated carbonyl occurred at δ 179.2 and the chemical shifts of the two carbons attached to hydroxyls were noted at δ 159.5 and 152.6. Based on these spectral evidences and comparison with reported data, the compound was identified as 7-hydroxynoracronycine (**2**).

The third acridone alkaloid isolated as yellow powder with m.p. 274-276 °C (m.p. 275 °C, [11]) was identified as atalaphyllidine (**3**) after comparison of its spectral data with literature data. The compound was previously reported to occur in *Atalantia monophylla*. The compound has been reported to be a good candidate as anticancer agent with potent antiproliferative activity against tumor cell lines but weak toxicity on normal cell lines [14].

## 3. Experimental

### 3.1. General

Melting points were determined on a Kofler hot plate and are uncorrected. IR spectra (KBr) were recorded on a Perkin-Elmer FTIR model 1725X spectrophotometer. UV spectra were obtained on a Shimadzu UV-2100 spectrophotometer. NMR spectra were run on a JEOL JNM CRX 400FT NMR spectrometer equipped with 5 mm ^1^H and ^13^C probes operating at 400 and 100 MHz, respectively, with TMS as internal standard. The MS were obtained with a Shimadzu GCMS-QP5050 spectrometer with Direct Induction Probe (DIP) using ionization induced by electron impact at 70 eV.

### 3.2. Plant Material, Extraction and Isolation

The plant was collected in Sabah, Malaysia in 2000 and a voucher specimen has been deposited at the Forest Department Sandakan, Sabah (SAN138652). The finely ground air-dried stem barks of *G. macrantha* (600 g) were immersed sequentially with hexane, chloroform and methanol (each lasted for 72 hours using 5 liters of each solvent) to give 1.32, 4.02 and 19.5 g of dark gummy semisolid extracts upon solvent removal. The hexane extract was chromatographed over silica gel and eluted with gradient of hexane, ethyl acetate and methanol to give 81 fractions of 100 mL each. Further chromatographic separation of fractions 19-27 and recystallisation with hot chloroform lead to isolation of macranthanine (**1**, 24 mg). Compound (**1**) was also isolated from the chloroform extract. Similarly, the methanol extract (12.5 g) was separated by column chromatography and eluted with gradient mixtures of hexane, ethyl acetate and methanol to give 96 fractions of 100 mL each. Fractions 30-37 were combined and further separated by column chromatography to give 45 sub-fractions of 50 mL each. 7-Hydroxynoracronycine (**2**, 118 mg) was obtained from sub-fractions 35-38 as yellowish solid. Fractions 56-66 from the earlier separation was similarly further separated by column chromatography to give atalaphyllidine (**3**, 108 mg).

### 3.3. Spectral Data

*Macranthanine* (**1**). UV λ_max_ (MeOH) nm (log ε): 303 (2.21), 239 (0.58); IR (KBr) ν_max_ cm^−1^: 3307, 2925, 1730, 1642, 1468, 1367, 1301, 1167, 1119; ^1^H-NMR (acetone-*d*_6_) and ^13^C-NMR (acetone-*d*_6_): see Table 1. EIMS m/z (rel. intensity): 375 [M]^+^ (22), 360 (75), 345 (10), 318 (6), 188 (15), 166 (6), 136 (5), 115 (5), 77 (5).

*7-Hydroxynoracronycine* (**2**). UV λ_max_ (MeOH) nm (log ε): 296 (2.18), 308 (2.71); IR (KBr) cm^−1^: IR ν_max_ cm^−1^: 3365, 2924, 1738, 1636, 1459, 1365, 1212, 1119; ^1^H-NMR (DMSO-*d*_6_) and ^13^C-NMR (DMSO-*d*_6_): see Table 1. EIMS m/z (rel. intensity): 323 [M]^+^ (26), 308 (100), 293 (20), 279 (4), 264 (6), 250 (5), 236 (6), 154 (37), 146 (17), 140 (6), 119 (4).

*Atalaphyllidine* (**3**). UV λ_max_ (MeOH) nm (log ε): 298 (1.29), 259 (2.43); IR (KBr) ν_max_ cm^−1^: 3389, 2963, 1644, 1479; ^1^H-NMR (500 MHz, DMSO-*d*_6_) δ: 1.39 (*s*, 6H, H-14 & 15), 5.68 (*d*, 1H, *J* = 9.15 Hz, H-12), 6.02 (*s*, 1H, H-2), 6.93 (*d*, 1H, *J* = 9.15 Hz, H-11), 7.12 (*t*, 1H, *J* = 8.05 Hz, H-7), 7.18 (*d*, 1H, *J* = 8.05 Hz, H-6), 7.61 (*d*, 1H, *J* = 8.05 Hz, H-8), 14.57 (*s*, 1H, 1-OH). ^13^C-NMR (125 MHz, DMSO-*d*_6_) δ: 27.7 (C-14 & C-15), 77.6 (C-13), 96.7 (C-2), 98.5 (C-4), 104.2 (C-9a), 115.1 (C-8), 115.7 (C-11), 117.1 (C-6), 120.2 (C-8a), 122.4 (C-7), 126.3 (C-12), 131.1 (C-5), 137.1 (C-10a), 145.7 (C-4a), 159.4 (C-3), 163.9 (C-1), 181.1 (C-9). EIMS m/z (rel. intensity): 309 (24), 294 (100), 293 (19), 264 (6), 236 (5), 180 (4), 154 (10), 147 (25), 133 (12), 110 (5).

## 4. Conclusions

Three acridone alkaloids were separated and identified from the stem bark of *Glycosmis macrantha* collected from Sabah, Malaysia. The compounds were obtained in crystallized form and two of them were new structures. The structures of the compounds were established by spectroscopic methods.
